# Does the Consecutive Interpreting Approach enhance medical English communication skills of Japanese-speaking students?

**DOI:** 10.5116/ijme.5abe.0eb5

**Published:** 2018-04-19

**Authors:** Hideki Iizuka, Alan K. Lefor

**Affiliations:** 1 Division of Languages and Humanities (English), Department of Premedical Sciences, Dokkyo Medical University, Japan; 2 Department of Gastroenterological and General Surgery, School of Medicine, Jichi Medical University, Japan

**Keywords:** Medical English, consecutive interpreting, prosody, shadowing, reproduction, Japan

## Abstract

**Objectives:**

To determine if the Consecutive Interpreting Approach enhances medical English communication skills of students in a Japanese medical university and to assess this method based on performance and student evaluations.

**Methods:**

This is a three-phase study using a mixed-methods design, which starts with four language reproduction activities for 30 medical and 95 nursing students, followed by a quantitative analysis of perfect-match reproduction rates to assess changes over the duration of the study and qualitative error analysis of participants' language reproduction. The final stage included a scored course evaluation and free-form comments to evaluate this approach and to identify effective educational strategies to enhance medical English communication skills.

**Results:**

Mean perfect-match reproduction rates of all participants over four reproduction activities differed statistically significantly (repeated measures ANOVA, p<0.0005). The overall perfect-match reproduction rates improved from 75.3 % to 90.1 % for nursing and 89.5 % to 91.6% for medical students. The final achievement levels of nursing and medical students were equivalent (test of equivalence, p<0.05). Details of lexical- and syntactic-level errors were identified. The course evaluation scores were 3.74 (n=30, SD = 0.59) and 3.77 (n=90, SD=0.54) for medical and nursing students respectively.

**Conclusions:**

Participants’ medical English communication skills are enhanced using this approach. Participants expressed positive feedback regarding this instruction method. This approach may be effective to enhance the language skills of non-native English-speaking students seeking to practice medicine in English speaking countries.

## Introduction

The United States Educational Commission for Foreign Medical Graduates announced that physicians applying for the United States Medical Licensing Examination starting in 2023 would be required to graduate from a medical school accredited through a formal process using globally accepted criteria. In response to this announcement, the Japan Society for Medical Education published “Basic Medical Education: Japanese Specifications, Global Standards for Quality Improvement” based on the 2012 version of the World Federation for Medical Education standards and guidelines.^1^

Since the evaluation criteria of the Basic Medical Education document includes items unattainable without the enrichment of medical English language education, the Japan Society for Medical English Education established guidelines to meet the new global standards.^2^ These guidelines include four language categories: Vocabulary, Reading, Writing, and Communication. There are three desired outcomes: 1) The ability to read and understand textbooks and literature in English, 2) the ability to interview and examine patients in English, and 3) the ability to conduct a presentation and discussion in English at an academic conference. Japanese authors were the most prolific contributors to Annals of Thoracic Surgery from 2004-2009 among authors from more than 27 “English as an International Language” countries and provided about 70% of manuscripts with 55% of the publications.^3^ This suggests that the goal of reading and writing of medical literature is achievable. However, fulfilling the other goals requires sophisticated verbal communication skills, and remains a significant challenge. As Guest points out, question/answer and discussion sessions at international medical conferences are the most anxiety-inducing aspect of giving an English-language presentation for Japanese doctors.^4^

Crystal illustrates three concentric circles regarding various roles that the English language serves in different countries: (a) the Inner Circle, where English is the primary language, (b) the Outer Circle, where English serves as a second language in a multilingual country, and (c) the Expanding Circle, where English is studied as a foreign language.^5^ Hawthorne argues that English language proficiency is a key factor in Australia, influencing employment outcomes for International Medical Graduates (IMGs) from Expanding Circle countries.^6^ Woodward-Kron et al. also reported that patient-centered interviewing by medical students in Australia whose first language is not English was hindered due to a limited repertoire of English grammar, vocabulary, and phonological phrasing for effective interaction, thus confirming the demand for a resource to help solve language difficulties.^7^ The literature describes formidable language barriers for IMGs or students from the Expanding Circle countries. Japanese medical students are no exception. However, an ideal teaching strategy to specifically solve such language difficulties has not been identified. To meet this challenge, we applied the Consecutive Interpreting Approach to Japanese medical English language education, which is described from an interdisciplinary perspective.

### The Consecutive Interpreting Approach

This approach is a sound-centric foreign language teaching method, in which shadowing for prosody acquisition is integrated with reproduction for enhancing speech awareness. Output activities in English place a considerable burden on cognitive processing in students who have very limited exposure to English in their daily lives, because such activities require various non-automated processes to be simultaneously performed. When a task demand exceeds a student’s ability, there is a high risk of the task ending in failure if the student does not receive any assistance. This concept is based on Vygotsky’s Zones of Proximal Development (ZPD) and demonstrates that when high-burden cognitive processing activities are performed, task demands need to be kept within the ZPD, that is, a zone just above the learner’s current developmental level.^8^ This can help achieve efficient capacity development, including foreign language manipulation. Therefore, Iizuka hypothesized that effective acquisition of foreign language would be enhanced if the cognitive burden associated with output is minimized and placed within the ZPD. To lower the cognitive burden, the Consecutive Interpreting Approach utilizes two strategies: shadowing and reproduction. Since the theoretical background of this approach has been described in detail, we briefly describe prosody, shadowing, reproduction, and consecutive interpretation that form the backbone of the approach and provide an overview of this instruction method.^9^

### Prosody

Prosody is a general term used to explain sound in language and includes accents, intonation, and pauses between sense-groups, as well as concatenation, reduction, and absorption of sounds. Someya stated that prosody accounts for 30% to 40% of meaning transmission in natural communication and that prosody acquisition is essential for the enhancement of verbal communication skills.^10^ Isochronism is a prosodic element that characterizes the English language. It refers to the tendency for one stressed vowel and the next stressed vowel to appear at equal intervals, regardless of the number of unstressed vowels between them. An example of isochronism is shown in Table 1. In contrast, Japanese is a language in which consonant + vowel units, called moras, tend to be accented equally.^11^ Thus, if the English sentences in Table 1 are read with Japanese prosody, all the syllables will be marked with ●. As shown in this example, Japanese students cannot hear unstressed syllables, which are read rapidly and weakly, unless they master the isochronism inherent in English. In addition, if English is pronounced in the rhythm of the Japanese language, it will introduce sounds that cannot be recognized by native English speakers. This gives rise to the following question: what methods are effective in acquiring English prosody, including isochronism?

**Table 1:** Example of isochronism in English

She	speaks	English
●	●	●
Students	are speaking	English
●	●	●
The students	have been speaking	English
●	●	●

Note: ● sections are accented and appear at equal intervals.

### Shadowing

Shadowing is a method of practice in which the learner tracks heard speech and repeats it as precisely as possible whilelistening attentively to incoming information.^12^ Someya stated that shadowing helps to enhance and nurture a prosodic sense.^10^ Therefore, prosody obtained through shadowing can solve many of the problems described in the previous section. However, shadowing still lacks a crucial element as a method for further improving verbal communication skills. Simply repeating a sound does not go beyond imitation and it does not work on conscious awareness of output to manipulate a foreign language. In a broader context, shadowing is classified as an activity that tends toward input. Reproduction activity using consecutive interpretation notes, as described below, can be a means of compensating for this lack.

### Reproduction using consecutive interpretation notes

In consecutive interpretation, the interpreter listens to the speaker (source language: SL) while taking notes, and consecutively renders each segment of the message in the target language (TL), based on the notes taken as shown in Table 2.

In the Consecutive Interpreting Approach (the instruction method used in this study), a part of this pattern is altered. After understanding the semantic content of an SL text, 1) students acquire prosody through shadowing and then 2) reproduce the SL text again in English both orally and in writing based on notes as described in Table 2. In other words, while minimizing a cognitive burden associated with the output, the Consecutive Interpreting Approach is intended to help students enhance awareness of output, including grammatical competence, and acquire verbal communication skills by continuously performing the activities described above in 1) and 2). This activity of reproducing the SL is, in a precise sense, different from speaking involving voluntary output of English. However, without this type of fundamental training of output, automatic speaking is not achieved.

**Table 2:** Consecutive interpretation and notes

Source Language (SL)	Notes	Target Language (TL)
The ban on Tamiflu does not apply to children under 10 because they are at risk of dying from the influenza that Tamiflu is known to treat effectively.	bn T × ap chi↓10 b/c rsk dyn flu T knw trt effc	SL is converted to the TL based on the notes.

Note: In this study, the notes are written in Japanese to nurture translation skills.

The present study has three objectives: (a) to measure changes in language reproduction rates among four sessions of writing reproduction activities, (b) to identify language areas this approach cannot support by carrying out error analysis of students' language reproduction, and (c) to review this instruction method from a detailed course evaluation given after the activities. We hypothesize that source language reproduction rate in reproduction activities will improve over time, with enhancement of verbal communication skills. This hypothesis is based on evidence found by Izumi that states students receiving both input and output had the greatest growth in linguistic skills.^13^ The Consecutive Interpreting Approach integrates both input (shadowing) and output (reproduction). By analyzing errors in reproduction, linguistic items that cannot be solved by this approach will be identified. The course evaluation encapsulates the participants’ view of this approach and may identify areas for further development.

## Methods

### Study design and participants

This study has a three-phase mixed-methods design: 1) a quantitative analysis of language reproduction rates for four consecutive reproduction activities, 2) a qualitative error analysis of students' language reproduction, and 3) a quantitative and qualitative detailed course evaluation after using the Consecutive Interpreting Approach. The mixed method allows us to evaluate how students' language reproduction rates change over time, identify languages areas that this instruction method does not cover, and review this approach from students' perspectives.

Participants included 30 first-year students from the School of Medicine and 95 first-year students from the School of Nursing, a total of 125 students at Jichi Medical University, Japan. There were absences in some classes, resulting in fewer total participants. The Japanese medical curriculum is based on a six-year program that starts after high school. Japanese nursing university education has a four-year curriculum for a Bachelor of Science degree in Nursing. Participants in this study have all studied English, starting in middle school. The participants’ reading skills are highly advanced but many of them are not well trained in speaking. Their English proficiency falls between Common European Framework of Reference for Languages (CEFR) B1 and B2 since we offer English entrance examinations with a level of approximately B1 for nursing and B2 for medical students. The Institutional Review Board of Jichi Medical University waived review of this study because of the nature of this research. The results of this study did not affect participants' grades and were used for research purposes only.

### Study setting

In both schools, this approach was implemented as a part of a required “Medical English Communication” course, which is offered once per week. In each class session, students are presented with a passage from a textbook, and they performed shadowing and oral/writing reproduction.^14^ Four consecutive weeks were allocated for this study enabling students to study four passages with this instruction method. Each passage was distributed weekly in ascending order in terms of word and syllable count. The passages included materials about Norwalk virus, Tamiflu, mad cow disease and eating disorders. As shown in Table 2, students were also given a set of “Consecutive Interpreting Notes” prepared by the instructor, for the reproduction activities.

### Procedure

#### 1) Shadowing

An audio compact disc (CD) of the passage read by a native English speaker is played for shadowing practice. Participants use the sound only, and no printed text is presented at this stage. Suzuki reported that shadowing of unknown texts, in which students attempt to guess the meaning of the content while shadowing, activates cognition and improves auditory comprehension significantly compared to the shadowing of a known text.^15^

#### 2) Source Language (English) Analysis

A printed version of the passage is distributed, and participants confirm the vocabulary and grammar items of the English sentences for semantic comprehension. This process corresponds to the grammar-translation method in a conventional English course.

#### 3) Parallel Reading and Shadowing

After comprehending the meaning of the passage, parallel reading and shadowing are performed while replaying the audio CD. According to the Second Language Acquisition definition, repeating the sound while looking at the text is called parallel reading. In this process, participants are prompted to clarify and acquire prosodic elements of the passage by comparing the sound and written texts. Participants alternate parallel reading and shadowing until they are able to perform shadowing thoroughly.

#### 4) Oral and Writing Reproduction

Consecutive Interpreting Notes (Table 2) are presented, and participants orally reproduce the passage from the notes. If reproduction is difficult, parallel reading and shadowing are repeated. After the oral reproduction activity, participants reproduce the passage in writing based on the notes. To quantify changes in verbal communication skills, the oral reproduction activity of each student could have been recorded and analyzed but was not done in this study due to a large number of participants.

**Table 3:** Perfect-match reproduction rates

Passage	School of Medicine			School of Nursing		
	n	M	SD	n	M	SD
1	30	89.5	7.3	90	75.3	19.7
2	28	89.5	14.1	93	78.0	22.4
3	29	90.1	9.1	88	85.0	18.8
4	29	91.6	11.3	92	90.1	17.1

Note: n=number of participants, M= mean; SD = standard deviation

### Data collection instrument

The participants’ writing reproduction activities were converted into a numerical score using a scoring sheet (see Appendix). On this sheet, participants compare sentences they have reproduced in writing with the corresponding sentences in the source language and count the number of perfectly matching words. Perfect match reproduction rates are calculated by (number of perfectly matching words)/(total number of words in the source language) x 100. This scoring sheet was also used to identify the sentence with the lowest reproduction rate among four passages for a qualitative error analysis of students' language reproduction.

To review this instructional approach from the participants' perspective, the standard university “Course Evaluation” form was used which includes four items (the essential points of the course were easy to understand, the material was easy to understand, the teacher’s speech was clear and easy to understand, and the content of the course was interesting and fostered motivation to learn) rated on a four-point Likert scale (strongly agree-four points, agree-three points, tend to disagree-two points, and disagree-one point). Students may also write free-form comments.

### Data analysis

SPSS Statistics Version 21 software (IBM Corporation, Armonk NY) was used for descriptive statistics and statistical analysis. The differences in perfect match reproduction rates among four reproduction activities were tested for significance using a repeated measures ANOVA and post hoc tests with the Bonferroni correction. Statistical significance for all tests was set at p<0.05. Test of equivalence using R version 3.3.4 (R Core Team 2016, R Foundation for Statistical Computing, Vienna, Austria) with alpha = 0.05, assuming equal variances, and equivalence bounds of d=-7.251 and d= +7.251 being significant, was also performed to evaluate equivalence of final achievement levels between the two schools.

**Table 4:** Errors in the sentence with the lowest reproduction rate

Sentence with the lowest reproduction rate			
The signs and symptoms of Norwalk virus infection are nausea, vomiting, diarrhea and stomach cramps.			
Lexical level		Syntactic level	
Source Language	Errors	Source Language	Errors
symptoms	synptoms, symptons, synpton	signs and symptoms	sign and symptom
nausea	nousea, nusia, neusea, nosia	signs and symptoms are	signs and symptoms is
vomiting	vormiting, vomitting		
diarrhea	diaria, diarrea, diarier		
stomach cramps	stomach clamps, stmack, crumps		

## Results

### Perfect-match reproduction rates

Repeated measures ANOVA with sphericity assumed analysis showed that the mean perfect-match reproduction rates of all participants (N=117) over four passages differed significantly between time points (F_(3, 348)_= 0.864, p< 0.0005). Post hoc tests using the Bonferroni correction revealed that this instruction method elicited a small increase in perfect-match reproduction rates from passage 1 to passage 2 (78.8 ± 18.5 % vs 80.7 ± 21.6 %, respectively), which was not statistically significant (p=1.000). However, reproduction rates of passage 3 increased to 86.3 ± 17.0%, which was significantly higher than the passage 1 reproduction rate (p= .011). Reproduction rates for passage 4 increased to 90.5 ± 15.9%, which was also statistically significant compared to passages 1 (p< .0005) and 2 (p< .0005) reproduction rates. This suggests that long-term training (1 month) based on the Consecutive Interpreting Approach elicits a statistically significant increase in perfect-match reproduction rates, but not after one week of training. Table 3 shows the descriptive statistics of perfect match reproduction rates of four passages for the School of Medicine and School of Nursing.

These results show that students in the School of Medicine maintained a high perfect match reproduction rate, which increased slightly from 89.5 to 91.6% over the duration of the course. In contrast, the perfect match reproduction rates for students in the School of Nursing increased from 75.3% to 90.1%. This suggests that using this instruction method improves perfect-match reproduction rates, especially for students in the School of Nursing. Students from both schools achieved over 90% reproduction rates by the time they reached passage 4. A test of equivalence was performed with alpha=0.05, assuming equal variances, and equivalence bounds of d=±7.251 being significant. These results indicated that students from the School of Nursing achieved a reproduction rate equivalent to students from the School of Medicine for the final passage (p=.0198).

**Table 5:** Participant free-form comments

Quote	
1	“I find that creating English sentences in my head based on the notes and speaking English with a conscious awareness of the sound flow are essential to have a good command of English. By practicing shadowing repeatedly, I felt my English sentence became more fluid and fluent”. (Medical student, male)
2	“The experience of the unique English learning method was very exciting to me. I am very happy because I realized I am able to create English sentences using only notes faster and faster each time”. (Medical student, female)
3	“By creating English sentences from notes in the class, I not only memorized English sentences but also thought actively to create English sentences when I forgot the memorized ones. It is nice that I realize my English proficiency has improved”. (Medical student, male)
4	“The practice of shadowing and training to create speech from notes in class was very new and challenging to me. I was able to work on every task diligently”. (Medical student, male)
5	“The speaking course was very good since I had a strong feeling of active participation in the course”. (Medical student, female)
6	“I have not only improved my English reading skills but also, I feel even as if English has become part of myself. I enjoy doing presentations in English”. (Nursing student, female)
7	“The speaking course was very nice because I was able to reinforce my speaking skills which had been insufficient in conventional English learning”. (Nursing student, female)

### Errors in the sentence with the lowest reproduction rate

Among all sentences in passages 1 to 4, the sentence with the lowest reproduction rate is sentence 4 in passage 1 for students in both schools. The reproduction rate of this sentence was 80.2% for students in the School of Medicine and 61.3% for those in the School of Nursing. Table 4 shows this sentence and examples of errors grouped into lexical and syntactic levels.

### Course evaluation

Students rated the course at a mean score of 3.74 (N = 30, SD = 0.59) in the School of Medicine and 3.77 (N= 90, SD = 0.54) in the School of Nursing. Free-form comments were extracted, and a sample is shown in Table 5.

## Discussion

### Perfect match reproduction rate

The participants’ ability to accurately reproduce English sentences was improved gradually using the Consecutive Interpreting Approach, thus supporting the study hypothesis that the source language reproduction rate improves over time, resulting in enhancement of verbal communication skills. Comparing the results between the Schools of Nursing and Medicine is of great interest, and there are several possible explanations for the differences observed. Identical passages were used in both study groups, and it may be necessary to use different passages to observe a change in results over time for students in the School of Medicine. As shown in Table 3, students in the School of Nursing improved their perfect-match reproduction rates more than medical students, indicating that this instruction method brings about more positive outcome for CEFR B1 level students. According to Pawley and Syder, the utterances one produces are composed not only of sentences constructed with the help of syntactic rules but of sequences of words or phrases retrieved from memory as one unit called formulaic language.^16^ The reason for the better performance achieved by nursing students could be attributed to the fact that they encountered more syntactic rules and formulaic languages which were new to them and accumulated more of them than medical students did throughout this study.

### Error tendencies

As shown in Table 4 students performed poorly in the spelling of medical-related words including diarrhea, nausea, vomiting, and stomach cramps. JACET 8000 is a lexicon reference including 8,000 words necessary for Japanese people to facilitate international communications.^17^ The Japanese Association of College English Teachers (JACET) selected those words as part of an academic project through an analysis of the British National Corpus (BNC) including 100 million words and a sub-corpus collecting English data of high importance for Japanese people. Analysis of the words in Table 4 with the JACET 8000 analysis program “v8an” shows that these words exceed Level 8, which is the final attainment target for general word learning for Japanese English learners.^18^ These words are categorized as technical terms.

However, based on the students’ spelling, they acquired almost correct prosody except for some spellings such as “stomach clamps”. Therefore, the oral reproduction level is thought to be acceptable. The syntactic-level errors were typically found in the use of singular and plural nouns and in subject-verb agreement especially when the subject was a plural noun. These results suggest that students should be taught to become more aware of subject-verb agreement in oral reproduction as well as reinforce their technical terms by use of medical lexicons. These findings support the study hypothesis that by analyzing reproduction errors, linguistic items that cannot be solved by this approach can be identified.

### Course evaluation

The perfect match reproduction rates in the School of Medicine indicate a ceiling effect, suggesting that the task may have been too easy for the medical students. However, their free-form comments including “very exciting” (quote 2, Table 5), “very new and challenging” (quote 4, Table 5), “strong feeling of active participation in the course” (quote 5, Table 5), reveal that the students from School of Medicine participated in the course actively. The concern that the task given in the course was too easy is dispelled to some extent and supports the continued use and application of this approach for enhancing medical English communications for students in both schools.

### Limitations

There are acknowledged limitations to this study. It was unexpected that results for students in the School of Medicine do not show a significant difference over time for perfect match reproduction rate, in contrast to what was shown for students in the School of Nursing. This suggests that future research may require the use of different source materials for students in the two schools. In addition, the sample size in the School of Medicine was smaller and may need to be expanded to demonstrate a difference. With the sample size used in this study, writing reproduction was analyzed and evaluated. It was not possible to assess the actual verbal reproduction rates. Future studies should include an evaluation of verbal reproduction rates.

Error analysis presents some problems to be solved including reinforcement of medical terms and idioms, and the stream of consciousness during oral reproduction, specifically in sentence construction with a conscious awareness of subject-verb agreement. Further studies are underway to solve the challenges.

## Conclusions

Changes in English language reproduction skills of participants studying medical English in both the School of Medicine and the School of Nursing were evaluated quantitatively and qualitatively, based on the Consecutive Interpreting Approach. The results show that the source language reproduction rates improved with each iteration, especially in the School of Nursing and their final achievement level was equivalent to that in the School of Medicine. The course evaluation and free-form comments from students in the School of Medicine reveal that students welcomed this approach. These results indicate that the Consecutive Interpreting Approach is effective in enhancing the verbal communication skills of Japanese medical and nursing students, in medical English. This instruction method may apply to students in other Expanding Circle countries. We will continue to improve strategies to advance medical English education to meet the criteria for international accreditation not only for students in Japan but also for students or IMGs in the Expanding Circle countries.

### Acknowledgments

The present study was supported by a Grant-in-Aid for Scientific Research (C) for 2015-017 (15K02960) from the Japan Society for the Promotion of Science.

### Conflict of Interest

The authors declare that they have no conflict of interest.

## Supplementary material

Supplementary file 1 Appendix. The checklist scores
