# Supplementary material for: Does the Consecutive Interpreting Approach enhance medical English communication skills of Japanese-speaking students?
Source: Int J Med Educ. 2018 Apr 19;9:101–7. doi: 10.5116/ijme.5abe.0eb5 (PMC5969989; doi:10.5116/ijme.5abe.0eb5)
Supplement: Supplementary file 1 — Appendix. The checklist scores [file ijme-9-101-S1.pdf]

Appendix

The checklist scores

Sentences		○	△	×	?
1	Tamiflu is an oral anti-viral drug for the treatment of influenza A or B.	14			
2	Dozens of children under the age of 10 and teenagers have suffered mental or neurological disorders, including abnormal behavior, after taking Tamiflu.	22			
3	Thus, the Health, Labor and Welfare Ministry issued emergency instructions to prohibit doctors from prescribing it to patients aged 10 to 19.	22			
4	The ban on Tamiflu does not apply to children under 10 because they are at risk of dying from influenza that Tamiflu is known to treat effectively.	28			

Instructions

(i) Enter English sentences you were able to write down during Writing Reproduction activity in the column below the original English sentences.

(ii) (ii) Compare your English sentences with the original ones, and then enter the numbers of ○, △, ×, and ? of each line in the right box in the manner described below.

○… Word perfectly matching with the text above
△… Word very close to the original one or with a small spelling error
×… Word making no sense (Do not count the word you were not able to write down.)
?… Word difficult to determine as △ or ×
